# The Application of Head-Up Tilt Test to Diagnose Hemodynamic Type of Orthostatic Intolerance in Children Aged Between 3 and 5 Years

**DOI:** 10.3389/fped.2021.623880

**Published:** 2021-03-03

**Authors:** Runmei Zou, Shuo Wang, Fang Li, Ping Lin, Juan Zhang, Yuwen Wang, Yi Xu, Cheng Wang

**Affiliations:** ^1^Department of Pediatric Cardiovasology, Children's Medical Center, The Second Xiangya Hospital, Central South University, Changsha, China; ^2^Jishou University School of Medicine, Jishou, China

**Keywords:** orthostatic intolerance, neurally mediated syncope, head-up tilt test, children, hemodynamic type

## Abstract

**Objectives:** The head-up tilt test (HUTT) is a useful tool to assess autonomic function and to reproduce neurally mediated reflex. In this study, we evaluated the use of HUTT in pediatric patients aged 3–5 years with orthostatic intolerance.

**Materials and Methods:** The medical history and HUTT records of 345 (180 males, aged from 3 to 5 years) cases of patients who complained of symptoms of orthostatic intolerance and who visited the Syncope Ward, Children's Medical Center, The Second Xiangya Hospital, Central South University from January 2003 to December 2019, were reviewed retrospectively.

**Results:** Seventy-nine (22.9%) cases had positive responses to complete HUTT (basic HUTT and sublingual nitroglycerin HUTT), while 29 (8.4%) cases had positive responses if only basic HUTT was performed. Sublingual nitroglycerin provocation significantly increased the positive rate of the test (*x*^2^= 27.565, *P* < 0.001). The most frequent hemodynamic response to HUTT was vasoinhibitory type vasovagal syncope (12.2%), Syncope (28.7%), and dizziness (22.6%) were the most common symptoms. Eight cases discontinued the test due to intolerable symptoms without severe adverse events occurring.

**Conclusions:** HUTT was safe and well-tolerated and could be used to diagnose the hemodynamic type of orthostatic intolerance in children aged 3–5 years.

## Introduction

Orthostatic intolerance is common in children and adolescents, and is defined as individuals who have difficulty in tolerating an upright posture and who present signs and symptoms of transient loss of consciousness, fatigue, headaches, lightheadedness, visual disturbances, sweating, vomiting, nausea, abdominal pains, and so on when standing (1). These symptoms and signs can be abated when returned to the supine position. The mechanism involves an inability to maintain adequate venous return to the heart due to venous pooling, primarily in the lower body, during standing. Orthostatic intolerance can be diagnosed as postural tachycardia syndrome (POTS), neurocardiogenic syncope, orthostatic hypotension, and vasovagal syncope (2). A comprehensive history, physical examination, standing test, and electrocardiogram (ECG) are necessary for diagnosis.

The head-up tilt test (HUTT) is an important tool to assess autonomic function and is used for differential diagnosis of transient loss of consciousness and orthostatic intolerance in adults and children (3, 4). HUTT can be used to diagnose the hemodynamic type of orthostatic intolerance (5). HUTT data in pediatric patients under the age of 6 years are limited. In this study, we evaluate the clinical use of HUTT in children aged between 3 and 5 years with orthostatic intolerance.

## Methods

### Participants

We retrospectively reviewed 345 (180 males, aged between 3 and 5 years) cases of patients who complained of orthostatic intolerant symptoms of syncope, headaches, dizziness, visual disturbances, sweating, vomiting, nausea, abdominal pains, and so on when standing upright, and who visited the Syncope Ward, Children's Medical Center, The Second Xiangya Hospital, from January 2003 to December 2019. The demographic and clinical data including HUTT records were reviewed. Heart, pulmonary, cerebral, and other system diseases were excluded after an initial evaluation consisting of history, physical examination, baseline laboratory testing, ECG, Holter ECG, echocardiography, chest X-ray, electroencephalogram, and cranial CT or MRI. According to the age range, patients were divided into three groups: group 3–4 referred to those aged above or equal to 3 and <4 years, group 4–5 referred to those aged above or equal to 4 and <5 years, group 5–6 referred to those aged above or equal to 5 and <6 years.

### HUTT Protocol

The HUTT consisted of two stages: basic HUTT and sublingual nitroglycerin HUTT. The protocol had been carried out according to a previous study (6, 7). HUTT was approved by the Ethics Committee of The Second Xiangya Hospital, Central South University. Informed consent was obtained directly from all the subjects or their guardians. The test was performed after overnight fasting between 8:00 a.m. and 12:00 p.m.. The subjects were asked to lay still for 10 min, and then basic heart rate (HR), blood pressure (BP), and ECG were recorded. Subjects were tilted at 60° head upward, and HR, BP, and ECG were recorded continuously until either 45 min duration, or the development of syncope or intolerable near syncope symptoms (basic HUTT). If syncope occurred, patients were rapidly placed in the supine position. If the subjects did not develop syncope or presyncope, they underwent nitroglycerin stimulated HUTT. A tilted posture was maintained, subjects were medicated with nitroglycerin, and HR, BP, and ECG were recorded for 20 min or until syncope or presyncope occurred.

Positive responses to HUTT included vasovagal syncope (VVS), postural tachycardia syndrome (POTS), orthostatic hypotension (OH), and orthostatic hypertension (OHT) (6). VVS was defined as the development of syncope or presyncope accompanied by hypotension (systolic BP ≤ 80 mmHg and/or diastolic BP ≤ 50 mmHg, or over 25% decrease in mean blood pressure), bradycardia (HR < 75 bpm), or cardiac arrest >3 s. VVS was further classified into three responses: vasoinhibitory type VVS (significant reduction in BP but insignificant change in HR), cardioinhibitory type VVS (significant reduction in HR but insignificant change in BP), and mixed type VVS (significant reduction both in BP and HR). POTS was defined as dizziness, chest distress, headaches, palpitation, and pallor with an increase in HR ≥ 40 bpm within 10 min of HUTT. OHT was defined as (within 3 min of HUTT) orthostatic intolerance symptoms and an increase in systolic BP ≥ 20 mmHg, and/or diastolic BP increments ≥ 25 mmHg without an obvious change in HR. OH was defined as (within 3 min of HUTT) orthostatic intolerance symptoms and a decrease in systolic BP ≥ 20 mmHg, and/or diastolic BP ≥ 10 mmHg.

### Statistical Analysis

Statistical analysis was performed by SPSS 17.0. Data were described as mean ± SD for normally distributed continuous variables, median (P_25_, P_75_) for non-normally distributed continuous variables, and percent prevalence for dichotomized variables. One-way analysis of variance (ANOVA) was used to analyze three or more normally distributed continuous variables. Manne-Whitney test was used to compare non-normally distributed continuous variables among groups. χ^2^ test, or Fisher exact test was used to compare dichotomized variables. *P* < 0.05 were considered statistically significant.

## Results

### Basic Characteristics of the Study Population

There were 38 subjects [22 (57.9%) males] aged between 3 and 4 years, 114 individuals [65 (57.0%) males] aged between 4 and 5 years, and 193 subjects [93 (48.2%) males] aged between 5 and 6 years. There were no significant differences in gender ratio, history duration, basic systolic, and diastolic blood pressure among the three groups, except for the fact that HR decreased, and body mass index increased with increasing age (*P* = 0.004 and *P* < 0.001, respectively) (Table 1).

**Table 1:** Basic characteristics of the study population.

**Age range**	**No. (%)**	**Male (%)**	**BMI (kg/m^**2**^)**	**Duration (mouth), median (P25, P75)**	**SBP (mmHg)**	**DBP (mmHg)**	**HR (bpm)**
3–4 Years	38 (11.0)	22 (57.9)	12.7 ± 5.7	6.5 (0.7, 12.0)	100.9 ± 14.6	62.6 ± 10.0	99.1 ± 14.8
4–5 Years	114 (33.0)	65 (57.0)	15.4 ± 5.5	2.0 (0.7, 6.3)	98.8 ± 13.1	61.6 ± 7.5	95.4 ± 13.4
5–6 Years	193 (55.9)	93 (48.2)	15.1 ± 3.3	2.0 (0.5, 12.0)	99.9 ± 9.8	61.2 ± 7.8	88.3 ± 14.0
Total	345 (100.0)	180 (52.2)	14.9 ± 4.5	2.0 (0.63,12.0)	99.7 ± 11.5	61.5 ± 7.5	91.9 ± 14.4
Statistic value	-	2.800	5.526	0.960	0.523	0.626	11.317
*P*	-	0.247	0.004	0.619	0.593	0.535	<0.001

*BMI, Body mass index; SBP, basic systolic blood pressure; DBP, diastolic blood pressure; HR, heart rate; bpm: beats per minute*.

### Presenting Symptoms in the Study Population

The most common symptoms were syncope (28.7%) and dizziness (22.6%), followed by sighing (11.9%), chest pain (8.4%), palpitation (6.4%), headaches (5.5%), and fatigue (1.2%). Other symptoms included visual disturbances, sweating, vomiting, nausea, and abdominal pains. The incidence of syncope decreased with increasing age (*x*^2^= 8.929, *P* = 0.012), whereas the incidence of dizziness increased with increasing age (*x*^2^= 6.910, *P* = 0.032) (Table 2).

**Table 2:** Presenting symptoms in the study population.

**Age range**	**Syncope**	**Dizziness**	**Sighing**	**Palpitation**	**Headache**	**Fatigue**	**Chest pain**	**Others**
3–4 years	18 (47.4)	4 (10.5)	5 (13.2)	4 (10.5)	0	0	1 (2.6)	6 (15.8)
4–5 years	35 (30.7)	21 (18.4)	18 (15.8)	9 (7.9)	7 (6.14)	2 (1.8)	7 (6.1)	15 (13.2)
5–6 years	46 (23.8)	53 (27.5)	18 (9.3)	9 (4.7)	12 (6.2)	2 (1.0)	21 (10.9)	32 (16.6)
Total	99 (28.7)	78 (22.6)	41 (11.9)	22 (6.4)	19 (5.5)	4 (1.2)	29 (8.4)	53 (15.4)
*x^2^* value	8.929	6.910	2.872	2.485	0.001	2.485	4.449	0.910
*P*	0.012	0.032	0.238	0.289	0.978	0.289	0.108	0.823

*Other symptoms referred to visual disturbances, sweating, vomiting, nausea, and abdominal pains*.

### HUTT Results

As shown in Table 3, 79 (22.9%) cases had positive responses to complete HUTT (basic HUTT and sublingual nitroglycerin HUTT), while 29 (8.4%) cases had positive responses if only basic HUTT was performed. Sublingual nitroglycerin provocation significantly increased the positive rate of the test (22.9 vs. 8.4%, *x*^2^= 27.565, *P* < 0.001). As shown in Table 4, hemodynamic types included 42 (12.2%) cases of vasoinhibitory type VVS, one (0.3%) case of cardioinhibitory type VVS, 14 (4.1%) cases of mixed type VVS, and 22 (6.4%) cases of POTS. There were no hemodynamic responses of OHT or OH.

**Table 3:** Number of the patients with positive or negative responses to the complete HUTT or only basic HUTT.

**Age range**	**Positive**		**Negative**		**Unfinished**	**Total**
	**Only basic HUTT**	**Complete HUTT**	**Only basic HUTT**	**Complete HUTT**		
3–4 years	7 (18.4)	11 (28.9)	30 (78.9)	26 (68.4)	1 (2.6)	38
4–5 years	9 (7.9)	23 (20.2)	99 (86.8)	85 (74.6)	6 (5.3)	114
5–6 years	13 (6.7)	45 (23.3)	179 (92.7)	147 (76.2)	1 (0.5)	193
Total	29 (8.4)	79 (22.9)^*^	308 (89.3)	258 (74.8)	8 (2.3)	345

*Complete HUTT referred that patients underwent two stages of HUTT (both basic HUTT and sublingual nitroglycerin HUTT)*.

* *Compared with only basic HUTT, P < 0.001*.

**Table 4:** Hemodynamic types in positive response to head-up tilt test.

**Age range**	**No**.	**VVS-V**	**VVS-C**	**mix-VVS**	**POTS**
3–4 years	38	4 (10.5)	0	0	7 (18.4)
4–5 years	114	12 (10.5)	0	4 (3.5)	7 (6.1)
5–6 years	193	26 (13.5)	1 (0.5)	10 (5.2)	8 (4.1)
Total	345	42 (12.2)	1 (0.3)	14 (4.1)	22 (6.4)
*x^2^* value	-	0.690	-	0.236	10.854
*P*	-	0.708	-	0.627	0.004

*VVS-V, vasoinhibitory type of vasovagal syncope; VVS-C, cardioinhibitory type of vasovagal syncope; mixed-VVS, mixed type of vasovagal syncope; POTS, postural tachycardia syndrome*.

### Compliance and Safety

Table 3 showed that eight (2.3%) cases did not complete the test due to intolerable sweating, abdominal pain, vomiting, and palpitations. However, none of these patients had severe adverse events during the HUTT process.

### Intervention Strategies in Case of Positive Responses

In case of positive responses, the patients were rapidly placed in the supine position. Strategies including keeping the airways open, oxygen therapy, and intravenous glucose infusion were taken when necessary. All the patients could recover completely within 5 min of rest in the supine position.

## Discussion

The medical history of 345 subjects aged between 3 and 5 years with orthostatic intolerance was reviewed in the present study. It was found that the most common symptoms were syncope and dizziness. Sublingual nitroglycerin provocation significantly increased the positive rate of the test compared with only basic HUTT. Vasoinhibitory type VVS was the most frequent positive response to HUTT. Eight patients did not complete the test due to intolerable symptoms, but none of these patients had severe adverse events during the HUTT process.

While upright, 70% of the blood volume was below the heart (8). Healthy individuals can maintain blood volume after standing due to autonomic and cardiovascular compensatory mechanisms. For patients with orthostatic intolerance, symptomatic hypotension occurs because of hypovolemia due to gravity and a time delay in sympathetic activation (9). BP and cerebral blood flow decrease inappropriately. BP can decrease by over 30% at 10–15 s after standing. Symptoms of lightheadedness and reflex tachycardia occur. With the increasing prevalence of orthostatic intolerance in pediatrics and the impairment of the quality of life for such patients, this area of research has drawn increasing attention in recent years.

HUTT has been used to reproduce neurally mediated reflex for years to investigate the heart rate and blood pressure adaptation to position change (10). A study previously reported the HUTT protocol in children aged between 4 and 18 years but did not include children aged below 4 years (11). In a retrospective study, 112 patients (mean age 15.6 years old) with orthostatic intolerance with abnormal HUTT results were included. The results suggested that headaches and syncope were the most frequent symptoms (46 and 29%, respectively) (4). In a single center, they reported that dizziness was the most common symptom in pediatric patients with POTS (age 5–18 years) (12). In our study, the most frequent symptoms were syncope (28.7%) and dizziness (22.6%), and 5.5% of cases had headache symptoms. The inconsistent results may be due to different populations and ages.

In our study, 79 (22.9%) cases had positive responses to complete HUTT (basic HUTT and sublingual nitroglycerin HUTT), while 29 (8.4%) cases had positive responses if only basic HUTT was performed, demonstrating that drug-free HUTT had a low positive rate. It is necessary to perform pharmacological provocation using isoproterenol intravenous infusion or sublingual nitroglycerin (13). A previous study suggested that isoproterenol intravenous infusion significantly increased the sensitivity of the test (from 28 to 45%) and was associated with a slight decrease in the specificity (from 93 to 86%) (14). Sublingual nitroglycerin challenge was safe in children with unexplained syncope with the specificity of 86% (15). In a meta-analysis, data demonstrated that nitroglycerine administration increased HUTT sensitivity but maintained a similar specificity in comparison to isoproterenol administration (16).

It is critical to identify the tilt angle to maximize the number of true positive responses and to minimize the number of subjects who are falsely assessed as positive responses. In a previous study, it is reported that the positive response appearance time was 15.1 ± 14.0 min, and the tilt angle was 60° in children (11). A meta-analysis confirmed that protocols with the tilt angle of 60° had a higher specificity associated with a lower sensitivity (16). Also, a tilt angle of 60° was utilized in our study.

HUTT is safe and well-tolerated. The previous study reported that the most common adverse events were the development of hypertension or tachycardia and intolerable flushing or nausea (17). In a previous study, 13 of 3,488 cases experienced transient aphasia during the HUTT process. The incidence of transient aphasia was higher in adults than in children (18). Approximately one-third of adult patients had some form of arrhythmic event during the HUTT process (19). Severe and rare cardiac complications such as atrial fibrillation was reported in the previous study (20). Eight of our patients discontinued the HUTT due to intolerable sweating, abdominal pain, vomiting, and palpitations. None of our patients suffered neurological and cardiovascular adverse events during the HUTT process.

Patients with orthostatic intolerance have difficulty in tolerating the upright posture and present symptoms and signs when standing, recovering when returning to the supine position. Our 345 patients were diagnosed with orthostatic intolerance according to the clinical manifestation. We further used HUTT to define the hemodynamic responses. For general patients with orthostatic intolerance, non-pharmacologic interventions including health education of avoiding prolonged standing, tilt training, and intake of water and salt were utilized to alleviate the orthostatic intolerant symptom. For patients with positive responses to HUTT, pharmacologic interventions such as midodrine hydrochloride or metoprolol should be used when appropriate (21). HUTT can not only diagnose the hemodynamic type but can also help with the selection of treatment strategies for orthostatic intolerance.

There were some limitations. In our study, 16.5% of cases presented VVS response with 12.2% of cases being of the vasoinhibitory type and 6.4% of cases presented a POTS response to HUTT. However, our study was a retrospective study. Data for healthy individuals aged between 3 and 5 years were not included. The specificity of HUTT for VVS and POTS could not be concluded. Otherwise, the standards for positive responses in children below 6 years of age were controversial.

In conclusion, the positive rate of the HUTT was 22.9% in pediatric patients aged between 3 and 5 years with orthostatic intolerance. The most common symptoms were syncope and dizziness. The most frequent hemodynamic response to HUTT was vasoinhibitory type VVS. Pharmacological provocation could improve the sensitivity of HUTT. HUTT was safe and well-tolerated for children aged below 6 years.

## Data Availability Statement

The original contributions presented in the study are included in the article/supplementary material, further inquiries can be directed to the corresponding author/s.

## Ethics Statement

The studies involving human participants were reviewed and HUTT was approved by the Ethics Committee of The Second Xiangya Hospital, Central South University. The informed consent was obtained from all the subjects directly or their guardians. Written informed consent to participate in this study was provided by the participants' legal guardian/next of kin. Written informed consent was obtained from the minor(s)' legal guardian/next of kin for the publication of any potentially identifiable images or data included in this article.

## Author Contributions

RZ was primarily responsible for the protocol development, patient enrollment, data collecting, preliminary data analysis, and for writing the manuscript. SW assisted with data analysis and critical revision for important content and edited the draft. FL, PL, JZ, and YW were responsible for completing the head-up tilt test. YX and CW supervised the design and execution of the study, checked the data analysis, contributed to the writing of the manuscript and provided final approval of the submitted manuscript. All authors have read and approved the final manuscript and assume full responsibility for its contents.

## Conflict of Interest

The authors declare that the research was conducted in the absence of any commercial or financial relationships that could be construed as a potential conflict of interest.
